# The Role of Adjuvant Radiotherapy for a Case of Primary Breast Sarcoma: A Plan Comparison between Three Modern Techniques and a Review of the Literature

**DOI:** 10.1155/2018/4137943

**Published:** 2018-04-17

**Authors:** Mariella Cozzolino, Caterina Oliviero, Barbara D'Andrea, Giuseppe Guglielmi, Giorgia Califano, Rocchina Caivano, Antonella Bianculli, Vincenzo Fusco

**Affiliations:** ^1^Department of Radiation Oncology, IRCCS CROB, Rionero in Vulture, Italy; ^2^Department of Radiation Oncology, University “Federico II”, Napoli, Italy; ^3^Department of Radiology, University of Foggia, Foggia, Italy; ^4^IRCCS “Casa Sollievo della Sofferenza”, San Giovanni Rotondo, Italy

## Abstract

A 65-year-old woman, affected by a malignant fibrous histiocytoma (undifferentiated pleomorphic sarcoma) of the left breast, presented to our department to receive the postoperative radiotherapy. In the absence of prospective and randomized trials and investigations on breast sarcoma irradiation in literature, due to the rarity of this pathology, the role of adjuvant radiotherapy remains unclear. To identify the best radiotherapy technique for this patient, three methods were compared: 3D conformal radiotherapy (3D-CRT), intensity-modulated radiation therapy (IMRT), and volumetric arc therapy (VMAT) or RapidArc® (RA). 50 Gy was prescribed to the chest wall and 66 Gy to the tumor bed. Three plans were designed, and target coverage, organs-at-risk sparing, and treatment efficiency were compared. IMRT and RA improved both target coverage and dose uniformity/homogeneity. Planning objective for the lung is always satisfied comparing the different techniques, but the volume receiving 20 Gy drops to 17% by RA compared to 3D-CRT. The heart volume receiving 30 Gy was 10% by IMRT, against 13% and 16% by RA and 3D-CRT. The monitor unit (MU) required by 3D-CRT was 527 MU, followed by RA and IMRT. Treatment time was similar with 3D-CRT and RA but doubled using IMRT. Although all three radiotherapy techniques offered a satisfactory solution, RA and IMRT offer some improvement on target coverage, dose homogeneity, and conformity for this particular case of breast sarcoma.

## 1. Introduction

Malignant fibrous histiocytoma (undifferentiated pleomorphic sarcoma) is considered to be the most common soft tissue sarcoma of middle and late adult life and characteristically affects the extremities and, less frequently, the retroperitoneum of elderly male patients. However, its occurrence in the breast is extremely uncommon, especially in patients with no history of radiation for a prior breast carcinoma or in cases that do not arise in association with a malignant phyllodes tumor [1]. Due to the low incidence, the therapeutic recommendations are difficult to establish with evolving techniques and limited patient numbers. Surgery is the mainstay of treatment for all breast sarcomas, with either wide local excision or total mastectomy [2–5]. There is an evidence that the tumor's size was predictive of local recurrence and overall survival [6, 7]. After surgical resection, radiation therapy (RT) should be used to improve local control in the cases in which the tumor is larger than 5 cm and in the cases with close or positive surgical margins. Postoperative RT has not been prospectively examined, and doses and treatment regimens have been infrequently described. Current knowledge is mostly based on numerous case reports and relatively small retrospective series; unlike epithelial breast cancer, there is no high level evidence to support a standard of care for primary and/or adjuvant therapy, but a trend of benefit for irradiated patients was reported, and therefore, adjuvant radiotherapy with higher doses was recommended for primary breast sarcomas, especially if the tumor is of larger size or high grade [8–10]. Johnstone et al. [11] observed excellent local control in patients who received adjuvant radiotherapy. McGowan et al. [12] reported that the cause-specific survival of the group, which received over 48 Gy radiation dose, was 91%; in comparison, the group which received no or less than 48 Gy radiation dose had a cause-specific survival of only 50%. Adjuvant radiotherapy decreased local failure from 34% to 13% in a series of 59 patients, according to a retrospective review of the M. D. Anderson experience [7], although this did not reach statistical significance probably due to the small number of patients.

The classical approach to the breast irradiation is the technique of two tangent fields, and also in the case of breast sarcoma, this technique is usually applied [13]. However, the advanced technologies such as intensity-modulated radiotherapy (IMRT) and RapidArc (RA) allow to administer higher doses, improving the dose conformity compared to 3D conformal radiotherapy, as already demonstrated for the treatment of breast cancer [14–17].

The aim is to present a case report of a left breast primary sarcoma, describing and comparing three modern radiotherapy techniques in order to identify the best approach for this patient. Moreover, we report a brief review of the literature about the role of adjuvant radiotherapy in the management of undifferentiated pleomorphic breast sarcoma.

## 2. Case Presentation

### 2.1. Patient, Structure Definition, and Dose Prescription

A 65-year-old woman, who presented a malignant fibrous histiocytoma (undifferentiated pleomorphic breast sarcoma) of the left breast and underwent the mastectomy, presented to our department to receive the postoperative radiotherapy. The lesion was greater than 5 cm with positive surgical margins and of high grade according to the NCI (National Cancer Institute) criteria for grading soft tissue sarcomas, with the impossibility to obtain a clear margin. No first-line chemotherapy was scheduled. According to the recommendation on the management by the ESMO Clinical Practice Guidelines, the postoperative radiation therapy should be administered with the best technique available, at a dose of 50–60 Gy, with fractions of 1.8–2 Gy, possibly with boosts up to 66–68 Gy, depending on the presentation and quality of surgery [18, 19]. So, two clinical target volumes were delineated on a series of CT slices by an experienced radiation oncologist: (i) the CTV50 which covered the entire left chest wall and (ii) the CTV66 defined by the tumor bed.

The CTV50 was delineated according to the breast cancer atlas for radiation therapy planning consensus definitions of the Radiation Therapy Oncology Group (RTOG) (available at http://www.rtog.org/CoreLab/ContouringAtlases/BreastCancerAtlas.aspx). The tumor bed was identified by surgical clips. The planning target volumes (PTV50 and PTV66) were obtained adding an all-round safety margin of 1.0 cm to the respective CTVs to account for setup uncertainties and respiratory motion [20]. PTVs were restricted to the skin cropping at 0.5 cm from the surface and to exclude the ribs. The heart, left and right lungs, and ribs were defined as OAR (organs at risk). Also, the contralateral breast was contoured.

**Figure 1:**
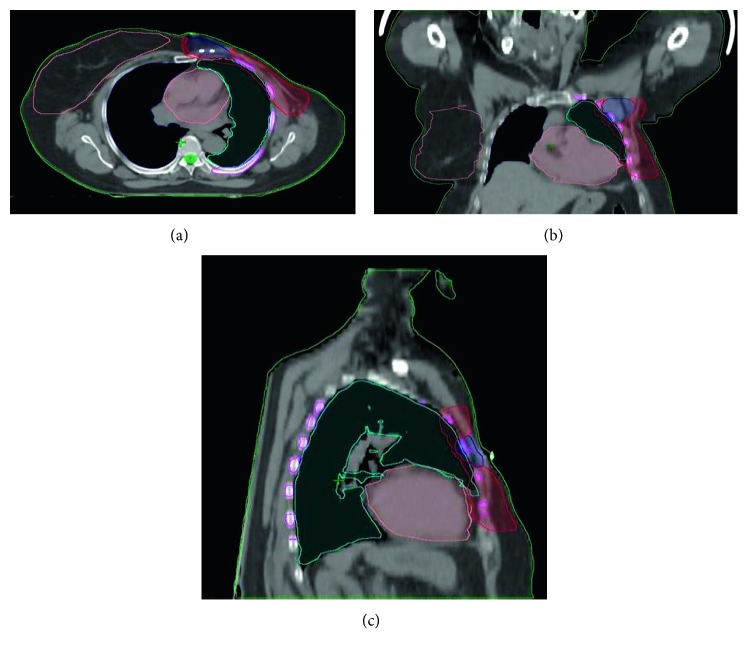
Axial, coronal, and sagittal planes of view from the CT data set of the patient, including target and OAR structures.

In Figure 1, a picture including three orthogonal planes from the CT data set of the patient is reported to appreciate the patient-specific anatomic complexity, which determines the complexity of the plan.

The 3D conformal radiotherapy (3D-CRT) treatment included two phases: a dose of 50 Gy was administered to the chest wall (2.0 Gy/fraction) during the first phase and a dose of 66 Gy to the tumor bed after the sequential boost (2.0 Gy/fraction). Unlike IMRT and RA, the plans were designed to deliver in a single-phase process (with simultaneous integrated boost (SIB)). The purpose of the simultaneous integrated boost (SIB) fractionation strategy proposed in this study is essentially to reduce the length of the treatment in order to improve patient satisfaction and clinical throughput. Limited investigations on SIB in breast irradiation are available in literature, proposing [16, 20–23] different schemes: 28 × (1.81 + 2.3) Gy, 31 × (1.66 + 2.38) Gy, or 25 × (2.0 + 2.4) Gy, for remaining breast and tumor bed targets. In all cases, the SIB plans with IMRT proved to have superior quality compared to sequential treatments, and the authors [22] proposed to consider SIB as the standard treatment for breast cancer. In the present study, it was adopted a further acceleration in the fractionation planning for 25 fractions (to keep treatment time limited to five weeks) of 2.0 Gy to the chest wall with a simultaneous integrated boost of 2.64 to the tumor bed. This fractionation has yet to be proven to be clinically acceptable; however, it does not impact the significance of comparative results.

All plans compared in this study were designed by the same planner on the Varian Eclipse treatment planning system (TPS) (version 8.6.10) with 6 and 15 MV photon beams from a Varian Trilogy accelerator equipped with a Millennium multileaf collimator (MLC) with 120 leaves. The anisotropic analytical algorithm (AAA) was used for all techniques, and the PB (pencil beam) algorithm was also employed for 3D-CRT plans. The progressive resolution optimizer (PRO) was used to optimize the RA plan. The dynamic sliding window method was used for the IMRT plan. The dose calculation grid was set to 2.5 mm for all plans.

The first phase of the 3D-CRT technique consisted of two no-coplanar tangential beams at 304° and 129° gantry angles, with collimator angle at 10° and 350°, respectively. Instead, the boost was achieved by three coplanar beams with gantry angles 300°, 115°, and 30° and collimator angles 280°, 80°, and 0°, respectively. Both for the first phase and for the boost, dynamic wedges were used to minimize the dose inhomogeneity.

The IMRT beam geometry consisted of 5 coplanar fields with the following gantry angles: 100°, 80°, 340°, 320°, and 300°, and the collimator angle was set at 30°. Dose rate of 400 MU/min was selected.

For the RA, a dual arc (one arc clockwise and another arc counterclockwise) was set up with a gantry angle ranging from 129° to 300° and a collimator angle of 30°, with a dose rate of 400 MU/min as the upper limit.

Throughout the IMRT and RA optimization, for all PTVs, plans aimed to achieve at least 95% of the PTV receiving more than 95% of the prescribed dose and a maximum lower than 107% to the 5% of the PTV66, while keeping the mean dose of each PTV as close as possible to the corresponding prescription. For the left lung, the conventional objectives were considered as acceptable, that is, the mean lung dose < 15 Gy and the volume receiving 20 Gy < 20–30% [24]. Similarly for the heart, the mean dose < 26 Gy and the volume receiving 30 Gy < 46% were set as planning objectives. For the ribs, the maximum dose < 70 Gy was set as an objective. Moreover, the planning strategy was also to minimize the mean dose to the contralateral lung.

All plans were normalized in order to guarantee that the dose received by 95% of the PTVs was greater than or equal to 95% of the prescribed dose (D95 > 95).

To assess the compliance with dosimetric objectives assigned, a comparison based on the cumulative dose-volume histograms (DVHs) was performed for all three treatment techniques. For the PTV50 and PTV66, the parameters D5% and D95% (doses received by the 5% and 95% of the target volume, resp.) were used as surrogate markers for the maximum and minimum doses. The mean dose (D_mean_) to both PTVs was also evaluated. The degree of conformity of the plans was defined as the ratio between the volume receiving at least 95% of the prescribed dose and the volume of the PTV. A conformity index (CI) equal to 1 corresponds to ideal conformation. If the CI is less than 1, the target volume is only partially irradiated, while a CI greater than 1 indicates that the irradiated volume is greater than the target volume including healthy tissues. Also, the homogeneity index (HI) was calculated. The HI was expressed by D5%−D95%/D_P_, where D5 and D95 are the doses to 5% and 95% of the target volume and D_P_ is the prescribed dose. The ideal value is zero when D5 and D95 are equal. The CI and HI were calculated for both PTV50 and PTV66.

The DVHs for OAR were also calculated and compared, using as evaluation tools the following parameters: the mean doses to the heart and lungs, the volume of the left lung receiving 20 Gy (V20), the volume of the heart receiving 30 Gy (V30), and the doses to 10% and 50% of the right lung (D10 and D50).

Monitor units (MUs) and delivery times were also analyzed for the three techniques. The delivery time was manually measured as the time from beam on to beam off.

Table 1 shows the DVH indices of the PTV50 and PTV66 in different treatment techniques. The planning objectives are also listed in order to assess the performances of plans. For 3D-CRT dose calculation, both AAA and PB algorithms were used, and the respective results are also listed in Table 1.

**Figure 2:**
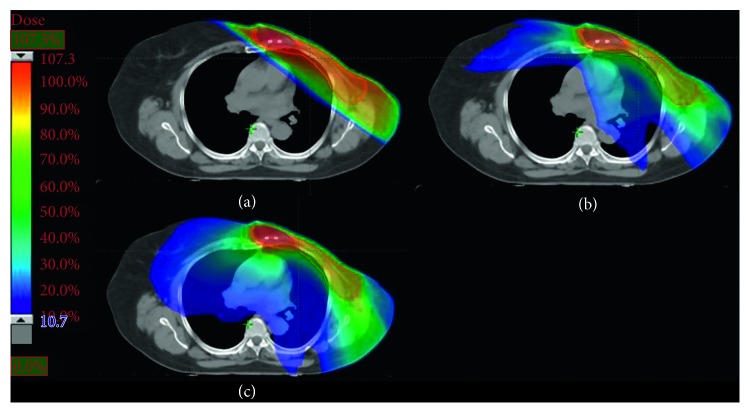
Axial dose distributions obtained by (a) 3D-CRT, (b) IMRT, and (c) RA.

**Figure 3:**
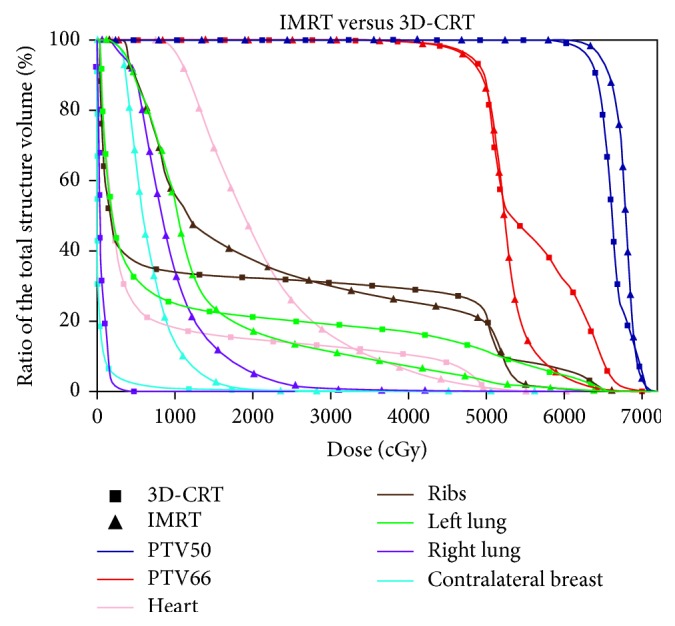
DVH comparison between IMRT and 3D-CRT for the target and OAR.

**Figure 4:**
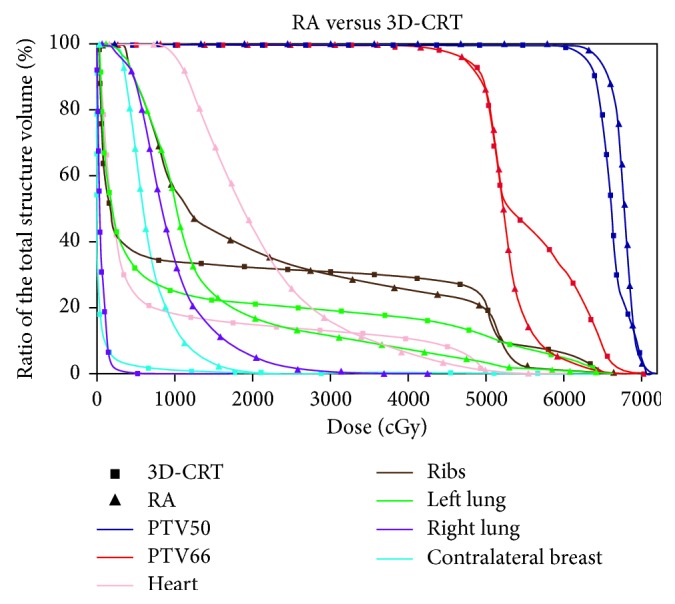
DVH comparison between RA and 3D-CRT for the target and OAR.

**Figure 5:**
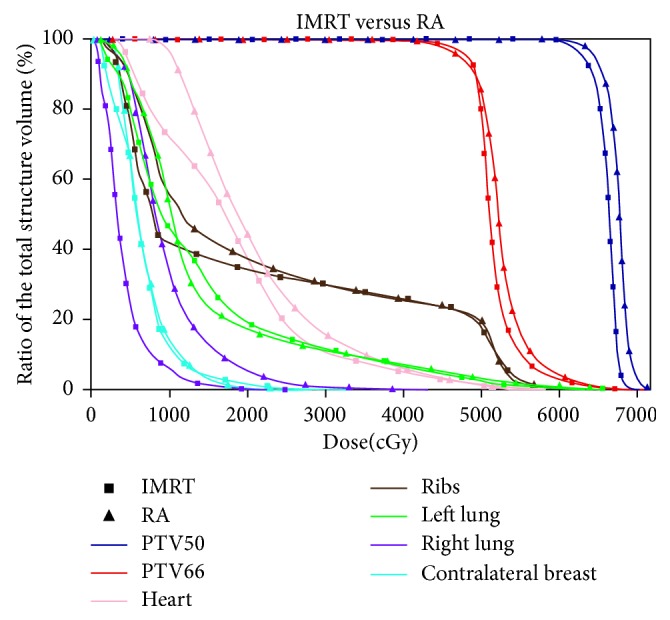
DVH comparison between IMRT and RA for the target and OAR.

Both RA and IMRT plans result in a better target coverage compared to the 3D-CRT, accompanied with an increased uniformity and homogeneity. The highest values of D95% for PTV50 and PTV66 are obtained by IMRT and RA, respectively. Globally, the IMRT achieves the best results. As shown in Table 1, the dosimetric differences between AAA and PB algorithms for 3D-CRT plans are not substantial. A picture of the axial dose distributions with the three techniques is shown in Figure 2. The comparison between three DVHs obtained by 3D-CRT, IMRT, and RA is shown in Figures 3–5.

The DVH parameters for OAR are also summarized in Table 1. The constraints set for the left lung are always satisfied by all the techniques, but the volume receiving 20 Gy decreases using RA and IMRT. Otherwise, the lowest mean dose to the ipsilateral lung is observed with the conformational technique. The mean doses to the heart increased with IMRT and RA, compared to 3D-CRT. The V30 of the heart obtained with IMRT is the lowest. There is an increased volume of the contralateral lung exposed to low doses both in the IMRT and RA plans. Also for the OAR, the dosimetric differences between AAA and PB algorithms for 3D-CRT plans are not substantial. Measurement of delivery time results in the lowest treatment time for RA (≈1.5 min) closely followed by 3D-CRT, whereas IMRT was somewhat time-consuming. The lowest number of MU required by 3D-CRT is 527, followed by RA and IMRT. Detailed results are given in Table 2.

The patient received radiation therapy in 2013 by 3D-CRT. After 3 years of follow-up, the patient remained disease free, and no evidence of local or systemic disease was detected. In 2016, bowel metastasis was detected with 18F-FDG PET and later confirmed with biopsy. Actually, the patient was treated with chemotherapy.

## 3. Discussion

Primary undifferentiated pleomorphic sarcoma arising de novo in the breast is extremely rare [25]. Qiu et al. summarized that 65 cases have been reported until 2013 [26]. Our literature review reveals that, to date, there are only five other cases presented [27–31]. Moreover, undifferentiated pleomorphic sarcoma in the breast is an aggressive, fast-growing tumor with a high rate of local recurrence (44%) and distal metastases (42%) [1], but the therapeutic recommendations are difficult to establish with evolving techniques and limited patient numbers. The most important treatment element in maximizing disease-free survival and overall survival is complete surgical removal of the tumor [32–36], although a standard surgical practice is still not known, owing to the rarity of this tumor. In the absence of prospective and randomized trials, response rates to systemic therapies remain poor [37]. Some authors suggest that postoperative radiation may play an important role in reducing local recurrence and should be considered for patients in whom surgical margins are not adequate or microscopically involved [38, 39]. However, the impact of RT on overall survival remains uncertain [40]. In one large study, patients with high-grade MFH of the extremities who underwent excisional surgery followed by postoperative RT experienced a 10-year relapse-free survival of 62% and an overall survival rate of 80%, certainly representing improved rates over historical reports [41]. Although further research is needed to better define the role of RT, the current NCCN guidelines state that adjuvant therapy should be considered on an individual case basis [19]. Radiation therapy should be given as an adjuvant to surgery only for primary intermediate- to high-grade breast sarcomas and a size larger than 5 cm. Recent review studies [2, 42, 43] proposed to treat patients according to the clinical practice guidelines in use for soft tissue sarcomas with a multidisciplinary team approach necessitating surgeons, pathologists, radiotherapists, and medical oncologists to improve overall survival. The treatment of a breast sarcoma must assure primarily an efficient target coverage with high prescribed doses to provide a better local control because even after radical surgery, local failures are common. This implies an appropriate prescription dose with at least 60 Gy to the tumor bed. The purpose of the present investigation was to assess, for a woman with this rare pathology, the quality of two advanced treatment techniques compared to the conventional radiotherapy, quantifying the DVH variations between the different solutions, and to appraise logistic aspects as treatment efficiency. Previously, the breast irradiation with advanced techniques was investigated, and the results [44] showed that, in certain cases, IMRT or RA/VMAT is definitely beneficial compared to conventional conformal approaches. The benefit of these advanced techniques was mainly demonstrated for a particularly complex and rare case as well as for bilateral breast cancer, left-sided breast cancer, small breast size, and funnel chest [16, 45, 46]. Also, for the postmastectomy irradiation, newer strategies such as IMRT were shown to improve significantly the dose distributions to the chest wall, maximizing dose homogeneity and conformity [47]. The data shown here suggest that all techniques are satisfactory, although RA and IMRT offer some improvement on target coverage, compared to the conformal radiotherapy. Comparing the DVH of the 3D-CRT to that of RA or IMRT, as shown in Figures 3 and 4, a substantial improvement of the dose homogeneity and conformity is clear. The best target coverage for the chest wall and tumor bed was achieved by using IMRT and RA, respectively (Figure 3, Table 1). Moreover, the conventional tangential field technique achieved fair target coverage only at the cost of delivering doses > 20 Gy to relatively large volumes of the ipsilateral lung, while similar better sparing of the ipsilateral lung was obtained using both RA and IMRT. Instead, the mean doses to the heart and contralateral lung were higher using RA compared to IMRT, mainly due to the larger volume exposed to lower radiation (Figure 2). The higher doses received by the heart, compared with those obtained by 3D-CRT, would be justified by patient-specific anatomic complexity partially due to the surgery and quite unfavorable boost position (Figure 1). Recent studies [48–50] showed that the risk of cardiovascular disease increases with mean cardiac doses in the women who received external beam radiotherapy for breast cancer, and the innovations in treatment planning are therefore under investigation, including intensity modulation and various techniques for the control of respiratory motion, in order to reduce the dose received by the heart. At our department, breath-hold technique has been implemented only recently, so not available in 2013 for more investigation. Henson et al. [51] demonstrated that the radiation-related risks were larger in the third decade after exposure than during the first two decades. Nevertheless, the OAR issue is very different between breast cancer and breast sarcoma because the life expectancy is not so high. Many patients survive for many years after diagnosis and treatment for breast cancer, and therefore, the awareness was created to reduce the dose received by the heart, in the hope of minimizing the long-term morbidity and mortality associated with the treatment of breast cancer. Unfortunately, the prognosis of sarcoma is still poor, and the risk of local recurrence is very frequent. For these reasons, the target coverage is the main goal, while OAR sparing could be considered secondary.

Another important objective was to assess the treatment efficiency. Naturally, both RA and IMRT techniques entail an increment of MU compared to the standard tangential field solution. Anyway, the RapidArc treatment time was 55% shorter than IMRT, implying a reduction of the risk of intrafractional movements. In addition, RA allowed a strong reduction of MU compared to IMRT (by 54%). So, the delivery parameters confirmed that RA was more efficient compared to IMRT. A recent study [13] reported the case of a 37-year-old male patient who received external radiotherapy for a malignant fibrous histiocytoma. The patient was offered a postoperative RT because of inadequate surgical margins. A total dose of 50 Gy/25f with two tangential wedged fields by using 6 MV photons of a linear accelerator was given to his left chest wall. He remained alive and well after 42 months of follow-up. Instead, Liu et al. [28] presented a case of a patient with carcinosarcoma of the left breast (mucinous carcinoma and MFH). Combined modality treatment, which consists of 6 cycles of adjuvant chemotherapy followed by 50 Gy/25f to the whole breast and 10 Gy/5f to the tumor bed, brings at least 2 years of disease-free survival (DFS). Anyway, no detailed information about the irradiation techniques was reported in literature, and no previous paper showed the application of intensity-modulated radiotherapy in the case of breast sarcoma. So, according to the previous results, we believe that the increase in the number of cases in the literature will help and contribute to the embodiment of the therapeutic algorithm of the disease in question [13].

## Figures and Tables

**Table 1:** DVH parameters for PTV and OAR for each investigated technique, together with planning objectives.

Region of interest	Index	3D-CRT PB	3D-CRT AAA	IMRT AAA	RA AAA	Planning objective
PTV66	D95% (Gy)	63.5	63.2	63.0	65.5	≥62.7
	D5% (Gy)	70.0	70	68.0	70.0	≤70.6
	D_mean_ (Gy)	67.0	66.3	66.0	67.8	≈66.0
	CI	0.94	0.92	0.96	0.99	=1
	HI	0.10	0.10	0.07	0.07	=0

PTV50	D95% (Gy)	47.5	48.0	48.2	47.9	≥47.5
	D5% (Gy)	66.9	65.8	57.6	60.2	≤53.5
	D_mean_ (Gy)	55.7	55.7	51.5	53.3	≈50
	CI	0.92	0.94	0.96	0.95	=1
	HI	0.38	0.35	0.12	0.22	=0

Heart	V30 Gy (%)	11.0	13.0	10.1	15.4	≤46
	D_mean_ (Gy)	8.1	8.4	17.7	19.3	≤26

Left lung	V20 Gy (%)	21.0	21.2	18.0	17.4	≤20–30
	D_mean_ (Gy)	12.5	12.5	13.6	14.3	≤15

Right lung	D10% (Gy)	1.6	1.3	7.5	16.3	—
	D50% (Gy)	0.55	0.35	3.2	8.3	—
	D_mean_ (Gy)	0.81	0.80	4.0	9.6	—

3D-CRT = 3D conformal radiotherapy; IMRT = intensity-modulated radiotherapy; RA = RapidArc; D95% and D5% = doses received by the 95% and the 5% of PTV volume; D_mean_ = mean dose to the structure; V30 and V20 Gy = volumes of the structure receiving 30 Gy and 20 Gy; D10% and D50% = doses received by the 10% and 50% of the structure; CI = conformity index; HI = homogeneity index.

**Table 2:** Delivery parameters of each investigated technique.

Parameter	3D-CRT	IMRT	RA
MU	527	1447	658
*T* (min)	1.75	3.44	1.54

3D-CRT = 3D conformal radiotherapy; IMRT = intensity-modulated radiotherapy; RA = RapidArc; MU = monitor unit; *T* = delivery time.
